# Supplementary material for: Continuous dynamics of cooperation and competition in social decision-making
Source: Commun Psychol. 2025 Nov 24;3:170. doi: 10.1038/s44271-025-00348-w (PMC12644803; doi:10.1038/s44271-025-00348-w)
Supplement: Supplementary file 2 — Supplementary Information [file 44271_2025_348_MOESM2_ESM.pdf]

Supplementary Information

Continuous dynamics of cooperation and competition in social decision-making

Darius Lewen[1,2†], Vladyslav Ivanov[3†], Jonas Dehning[1,2†], Johannes Ruß[2], Anna Fischer[2], Lars Penke[2], Anne Schacht[2], Alexander Gail[2,3], Viola Priesemann[1,2‡*], Igor Kagan[3‡*]

[1] Max Planck Institute for Dynamics and Self-Organization, Am Faßberg 17, Göttingen, Germany.
[2] University of Göttingen, Wilhelmsplatz 1, Göttingen, Germany.
[3] German Primate Center – Leibniz Institute for Primate Research, Kellnerweg 4, Göttingen, Germany.

† These authors contributed equally.
‡ These authors jointly supervised this work.

* Corresponding authors: viola.priesemann@ds.mpg.de, ikagan@dpz.eu

This PDF file includes:

Supplementary text 1. Supplementary Methods, including Supplementary Tables S1 to S5
Supplementary Figures S1 to S6
Supplementary Movies S1 to S9
Supplementary text 2. Instructions for participants

1 Supplementary Methods

1.1 Optimal dyad strategy

Table S1 | Variables used in section 1.1, and their descriptions.

Variable	Description
A, B	Agent names.
$\vec{a}_i, \vec{b}_i$	Agent positions at the start of cycle i.
$\vec{s}_i$	Single target position at the start of cycle i.
$\vec{j}_i^A$	Predominantly blue joint target (higher payoff share for agent A) position at the start of cycle i.
$\vec{j}_i^B$	Predominantly orange joint target (higher payoff share for agent B) position at the start of cycle i.
D_S	Relevant distance for the single target.
D_J	Relevant distance for a joint target.
D	Shortest relevant distance.
w	Target-type weighting parameter, weights single vs joint targets.
$\vec{t}_{i+1}$	Target position updating rule.
$\vec{x}_{i+1}$	Agent position updating rule.
$\vec{y}_{i+1}$	Extended agent position updating rule with advantageous placement.
N	Total number of collection cycles.
Φ	Fraction of single targets collected.

To establish an analytical foundation, we derive the optimal strategy for a dyad that maximizes their joint payoff. We assume for simplicity agents with identical speed, making the Euclidean distances from agents' positions $\vec{a}$ and $\vec{b}$ to the targets $\vec{s}, \vec{j}^A, \vec{j}^B$ the sole determinant of the payoff per second for each target choice. Given that the collection process of a single target begins when the first agent reaches it, the relevant distance for single targets D_S is the minimum distance between the agents and the target:

$$D_S(\vec{a}, \vec{b}, \vec{s}) = \min(||\vec{a} - \vec{s}||, ||\vec{b} - \vec{s}||). \tag{1}$$

Conversely, for joint targets, the collection process starts when the last agent arrives, so the relevant distance D_J is the maximum distance between the agents and the target:

$$D_J(\vec{a}, \vec{b}, \vec{j}^X) = \max(||\vec{a} - \vec{j}^X||, ||\vec{b} - \vec{j}^X||). \tag{2}$$

To maximize the joint payoff, we assume the dyad always selects the target with the shortest relevant distance D:

$$D(\vec{a}, \vec{b}, \vec{s}, \vec{j}^A, \vec{j}^B, w) = \min \begin{cases} D_S(\vec{a}, \vec{b}, \vec{s}) \cdot (1 - w) \\ D_J(\vec{a}, \vec{b}, \vec{j}^A) \cdot w \\ D_J(\vec{a}, \vec{b}, \vec{j}^B) \cdot w \end{cases}, \tag{3}$$

where w is a target-type weighting parameter. When $w = 1/2$, both target types are equally weighted, approximating the optimal dyad strategy. For $w \neq 1/2$, we obtain a non-optimal strategy that approximates the best strategy among those collecting the same fraction of single targets (FST value).
To simulate this strategy, we define the initial agent positions at the center of the game field:

$$\vec{a}_0 = \vec{b}_0 = \begin{pmatrix} 1/2 \\ 1/2, \end{pmatrix} \tag{4}$$

and sample the initial positions of the three targets from the two-dimensional standard uniform distribution:

$$\vec{s}_0, \vec{j}_0^A, \vec{j}_0^B \sim \mathcal{U}^2_{[0,1]}. \tag{5}$$

We define the target position updating rule $\vec{t}_{i+1}$ as implemented in the game

$$\vec{t}_{i+1} = \begin{cases} \vec{u} \sim \mathcal{U}^2_{[0,1]} & \text{if } \vec{t}_i \text{ was collected} \\ \vec{t}_i & \text{otherwise.} \end{cases} \tag{6}$$

If a target is collected, its new position is sampled from the two-dimensional standard uniform distribution $\mathcal{U}^2_{[0,1]}$; otherwise, it remains unchanged. In the first analysis of the optimal strategy, we assume both agents share the same position at the beginning of a collection cycle, defining the agent position updating rule $\vec{x}_{i+1}$ such that they are always at the previously collected target's position at the start of each collection cycle:

$$\vec{x}_{i+1} = \begin{cases} \vec{s}_i & \text{if } \vec{s}_i \text{ was collected} \\ \vec{j}_i^A & \text{if } \vec{j}_i^A \text{ was collected} \\ \vec{j}_i^B & \text{otherwise.} \end{cases} \tag{7}$$

With these updating rules, we can simulate each dyad strategy and thus calculate the expected fraction of single targets Φ:

$$\mathbb{E}[\Phi|w] = \lim_{N\to\infty} \frac{1}{N} \sum_{i=0}^{N} \begin{cases} 1 & \text{if } \vec{s}_i \text{ was collected} \\ 0 & \text{otherwise.} \end{cases} \tag{8}$$

and the expected relevant distance:

$$\mathbb{E}[D|w] = \lim_{N\to\infty} \frac{1}{N} \sum_{i=0}^{N} D(\vec{a}_i, \vec{b}_i, \vec{s}_i, \vec{j}_i^A, \vec{j}_i^B, w), \tag{9}$$

which is proportional to the payoff per second (see the gray curve in Fig. 3c for simulation results).

To relax the initial assumption of agents always sharing the same position at the start of each collection cycle, we extend the agent position updating rule: after collecting a joint target, both agents inevitably share a similar position, therefore, the updating rule remains in this case unchanged. However, during a single target collection, the non-collecting agent can position itself advantageously for the next collection cycle. Thus, we introduce an extended agent position updating rule $\vec{y}_{i+1}$:

$$\vec{y}_{i+1} = \begin{cases} \hat{\vec{y}}_{i+1} & \text{if } \vec{s}_i \text{ was collected by the other agent, and} \\ \vec{x}_{i+1} & \text{otherwise.} \end{cases} \tag{10}$$

This advantageous placement $\hat{\vec{y}}_{i+1}$ minimizes the expected relevant distance:

$$\hat{\vec{y}}_{i+1} = \arg\min_{\vec{y}} \mathbb{E}[D|\vec{x}_{i+1}, \vec{y}, \vec{j}_i^A, \vec{j}_i^B, w]. \tag{11}$$

Note here that $\vec{x}_{i+1} = \vec{s}_i$ since the previous collection was a single target collection. The expected relevant distance equals the integral over all possible single target spawn positions:

$$\mathbb{E}[D|\vec{x}, \vec{y}, \vec{j}^A, \vec{j}^B, w] = \int_{[0,1]^2} D(\vec{x}, \vec{y}, \vec{s}, \vec{j}^A, \vec{j}^B, w) d\vec{s}. \tag{12}$$

Fig. 3c (black curve) illustrates the simulation results with advantageous placement, and Fig. 3d and Supplementary Fig. S1 depict the expected relevant distances for various free-agent placements.

By simulating strategies for various $w \in [0, 1]$, we establish a function that maps target choices to FST values, $w \mapsto \Phi$ (Supplementary Fig. S2). Inverting this function allows us to predict dyads' target choices based on their FST value Φ and the current game configuration $\vec{a}_i, \vec{b}_i, \vec{s}_i, \vec{j}_i^A, \vec{j}_i^B$. The subsequent section will delve deeper into modeling dyad strategies.

1.2 Modeling dyad strategies

Table S2 | Variables used in section 1.2, and their descriptions.

Variable	Description
j	Index of collection type. Possible values: 1 $\Leftrightarrow$ single target collection, 2 $\Leftrightarrow$ predominantly blue joint target collection, and 3 $\Leftrightarrow$ predominantly orange joint target collection.
$\hat{y}_j^{(i)}$	Predicted probability for target collection j in collection cycle i.
Softmax	Softmax function.
$\boldsymbol{\theta}$	Regression coefficient matrix.
$\vec{x}^{(i)}$	Covariates of collection cycle i.
$\vec{x}_d$	Covariate component containing the relevant distances for the single and joint targets. The collection cycle i is omitted for simplicity.
$\vec{x}_p$	Covariate component encoding the identity of the previously collected target, whether an invite is present, and towards which joint target the invite is directed. The collection cycle i is omitted for simplicity.
$\vec{x}_{p'}$	Covariate component encoding the identity of the target collected before the previous one. The collection cycle i is omitted for simplicity.
$\boldsymbol{\theta}_d$	Component of the regression coefficient matrix that weights the relevant distance of the single target against those of the joint targets.
d_0	Regression coefficient of the single target's relevant distance.
d_1	Regression coefficient of the joint targets' relevant distances.
$\boldsymbol{\theta}_p$	Component of the regression coefficient matrix encoding either (1) if there is no invite, the relative change in collection probability of the previously collected target, or (2) if an invite is present, the relative change in collection probability of the joint target towards which the invitation is directed.
p_0	Regression coefficient for the change in the collection probability of the single target relative to others if the previous target was a single target.
p_1	Regression coefficient for the change in the collection probability of a joint target relative to others if the previous target was this joint target.
p_2	Regression coefficient for the change in the collection probability of a joint target relative to others if an invite towards this joint target is present.
$\boldsymbol{\theta}_{p'}$	Component of the regression coefficient matrix encoding the relative change in collection probability of the target collected before the previously collected target.
p_3	Regression coefficient for the relative change in the collection probability of the target collected before the previous one. This coefficient accounts for both single and joint targets.
$\mathcal{L}$	Negative log-likelihood function of the generalized linear model (GLM). It is the Cross-Entropy loss.
N	Total number of collection cycles.
$y_j^{(i)}$	Binary encoding indicating whether target collection j occurred in collection cycle i.

For each individual dyad, we fit a generalized linear model (GLM) to obtain predictions $\hat{y}_j^{(i)}$ about the probability for each possible target collection $j \in \{1, 2, 3\}$ given the collection cycles i's covariates $\vec{x}^{(i)}$ and dyads fitted regression coefficients $\boldsymbol{\theta}$

$$\hat{y}_j^{(i)} = \text{Softmax}(\boldsymbol{\theta}^T \cdot \vec{x}^{(i)})_j, \tag{13}$$

where the softmax function is employed to transform the linear combination of covariates $\boldsymbol{\theta}^T \cdot \vec{x}^{(i)}$ into a probability distribution over the target choices $j \in \{1,2,3\}$

$$\text{Softmax}(\vec{x})_j = \frac{\exp(x_j)}{\sum_{k=1}^{3} \exp(x_k)}. \tag{14}$$

The covariates $\vec{x}^{(i)}$ for each collection cycle i consist out of three stacked components

$$\vec{x} = \begin{bmatrix} \vec{x}_d \\ \vec{x}_p \\ \vec{x}_{p'} \end{bmatrix}. \tag{15}$$

The first component $\vec{x}_d$ encodes the relevant distances to the targets as in section 1.1

$$\vec{x}_d = \begin{pmatrix} D_S(\vec{a}, \vec{b}, \vec{s}) \\ D_J(\vec{a}, \vec{b}, \vec{j}^A) \\ D_J(\vec{a}, \vec{b}, \vec{j}^B) \end{pmatrix}. \tag{16}$$

The second component $\vec{x}_p$ encodes the end result of the previous cycle as a one-hot-encoded vector. This encompasses: (1) which target was collected and if it was a single target, (2) whether there is an invite (as defined in section 1.3), and if there is an invite, then towards which of the two joint targets it is.

$$\vec{x}_p = \begin{cases} \begin{pmatrix} 1 & 0 & 0 & 0 & 0 \end{pmatrix}^T & \text{if } \vec{s} \text{ previously collected and no invite,} \\ \begin{pmatrix} 0 & 1 & 0 & 0 & 0 \end{pmatrix}^T & \text{if } \vec{j}^A \text{ previously collected,} \\ \begin{pmatrix} 0 & 0 & 1 & 0 & 0 \end{pmatrix}^T & \text{if } \vec{j}^B \text{ previously collected,} \\ \begin{pmatrix} 0 & 0 & 0 & 1 & 0 \end{pmatrix}^T & \text{if } \vec{s} \text{ previously collected and invite towards } \vec{j}^A, \\ \begin{pmatrix} 0 & 0 & 0 & 0 & 1 \end{pmatrix}^T & \text{if } \vec{s} \text{ previously collected and invite towards } \vec{j}^B. \end{cases} \tag{17}$$

The third and last component encodes, also as a one-hot-encoded vector, which target type was collected two cycles ago.

$$\vec{x}_{p'} = \begin{cases} \begin{pmatrix} 1 & 0 & 0 \end{pmatrix}^T & \text{if } \vec{s} \text{ collected prior to the previously collected, i.e., two cycles ago,} \\ \begin{pmatrix} 0 & 1 & 0 \end{pmatrix}^T & \text{if } \vec{j}^A \text{ collected prior to the previously collected,} \\ \begin{pmatrix} 0 & 0 & 1 \end{pmatrix}^T & \text{if } \vec{j}^B \text{ collected prior to the previously collected.} \end{cases} \tag{18}$$

Analogous to the covariates $\vec{x}$ the coefficient matrix $\boldsymbol{\theta}$ consists out of three stacked components

$$\boldsymbol{\theta} = \begin{bmatrix} \boldsymbol{\theta}_d \\ \boldsymbol{\theta}_p \\ \boldsymbol{\theta}_{p'} \end{bmatrix}. \tag{19}$$

Where the first component $\boldsymbol{\theta}_d$ weights the relevant distance of the single target against those of the joint targets similar as the weighting w in section 1.1

$$\boldsymbol{\theta}_d = \begin{pmatrix} d_0 & 0 & 0 \\ 0 & d_1 & 0 \\ 0 & 0 & d_1 \end{pmatrix}. \tag{20}$$

The second component $\boldsymbol{\theta}_p$ does change either the relative probability of the previously collected target to the others or, if an invite is present, changes the relative probability of the joint target towards the invite is

$$\boldsymbol{\theta}_p = \begin{pmatrix} p_0 & 0 & 0 \\ 0 & p_1 & 0 \\ 0 & 0 & p_1 \\ 0 & p_2 & 0 \\ 0 & 0 & p_2 \end{pmatrix}. \tag{21}$$

The last component $\boldsymbol{\theta}_{p'}$ allows for a dependence further into the past. It modulates the probability of the next target towards the identity of the target prior to the previous target:

$$\boldsymbol{\theta}_{p'} = \begin{pmatrix} p_3 & 0 & 0 \\ 0 & p_3 & 0 \\ 0 & 0 & p_3 \end{pmatrix}. \tag{22}$$

Note that here in this component, the model does not differentiate between single and joint targets. To obtain the coefficient matrix $\boldsymbol{\theta}$, we use the quasi-Newton BFGS algorithm to minimize the negative log-likelihood function

$$\mathcal{L} = -\sum_{i=1}^{N}\sum_{j=1}^{3} y_j^{(i)} \ln(\hat{y}_j^{(i)}), \tag{23}$$

where $y_j^{(i)}$ is a binary encoding whether target collection j happened in collection cycle i or not and $\hat{y}_j^{(i)}$ is, as defined previously, the corresponding predicted probability of this target collection. The resulting prediction accuracy for unseen data (visualized in Fig. 4a) is evaluated for all collection cycles of the stable period (minutes 10–40) with k-fold cross-validation ($k = 5$). With this model, we can not only predict the dyad's target choice but we do also obtain an estimate of the dyad's uncertainty about the target choice at the start of the collection cycle (as utilized in Fig.5b and c). We estimate the dyads uncertainty with the uncertainty of our model which is quantified by the Shannon entropy H of the model's target prediction:

$$H(i) = -\sum_{j=1}^{3} \hat{y}_j^{(i)} \log_2(\hat{y}_j^{(i)}). \tag{24}$$

1.3 Trajectory classification

Trajectory classification is performed by a procedural algorithm that assigns a trajectory class (see Fig. 5a for visualizations) to each collection cycle based on a set of predefined conditions. The thresholds used in these conditions are set manually, and the trajectory classes are evaluated in a predetermined order. If a condition is met, the corresponding trajectory class is assigned to the cycle, and the algorithm proceeds to the next condition only if the current one is not met.

The first trajectory class evaluated is the "Invitation" class. A collection cycle is classified as "Invitation" if it follows a single target collection in the previous cycle, during which the non-collecting agent positioned itself in an "inviting" manner, that is, near a joint target before the collection of the previous single target collection is completed. This inviting placement can be either on the joint target (on-target-invite) or nearby the joint target (nearby-target-invite), with the latter defined as a distance less than half of that from the other agent. If the joint target is collected subsequently, the cycle is classified as "Invitation" otherwise, it is classified as "Failed invitation". If the nearby-target-invite condition is met, a cycle is only classified as "Failed invitation" if the overall FST is below 2/3, otherwise, it is more probable that the placement near the joint target is a coincidence. If neither the on-target-invite nor the nearby-target-invite condition is met, the algorithm proceeds to evaluate the "Strongly curved" trajectory class.

A collection cycle is classified as “Strongly curved” if the fraction of the excess trajectory length due to curvature exceeds 0.32. If this condition is not met, the agents' trajectories are relatively straight and the algorithm proceeds evaluating the condition of the “Different targets” trajectory class.

To determine whether the two agents aimed for different targets or the same, the algorithm fits straight lines to their trajectories, which serves to (1) smooth out the motor noise and (2) extend the aim of the agent beyond the final position reached. For each agent, the algorithm checks (1) whether the agent moved towards a target and (2) whether this target is in proximity to the corresponding line (i.e., less than 10.14 cm). If a target meets these conditions, it is considered a potential candidate for the agent's aim. If multiple candidate targets exist, the algorithm selects the one closest to the agent's final position after weighting the distances from the agents to the candidates according to the overall FST. This results in a prediction of which target each agent aimed for. If these predictions differ, the cycle is classified as “Different targets”. Note that if both agents initially aimed at different joint targets, the collection cycle would not end, leading to curved trajectories, which is why different initial aims towards joint targets are subsumed under the “Strongly curved” trajectory class.

Finally, the algorithm checks whether one agent moves ahead towards the target at which both agents aim. If the difference in distance averaged over the collection cycle to the finally collected target exceeds 3.4 cm, the cycle is classified as “One ahead”. If none of the above conditions hold true, the cycle is classified as “Concurrent” by exclusion principle.

1.4 Joint payoff

Table S3 | Variables used in section 1.4, and their descriptions.

Variable	Description
R	Joint payoff.
N	Number of target collections / collection cycles.
r	Payoff per target (7 cents).
T	Block duration (20 minutes).
t_i^{acq}	Duration of the acquisition period in cycle i.
$\langle\rangle$	Mean over all cycles.
t^{col}	Duration of the collection period (1 second).
e_1, e_2	Approximation errors.
l_i	Limiting trajectory length in cycle i.
s_i	Mean agent speed on the limiting trajectory in cycle i.
l_i^C	Additional trajectory length due to curvature in cycle i.
d_i	Limiting distance in cycle i.
d_i^T	Distance between the previous and the next target in cycle i.
d_i^R	Distance reduction due to advantageous placement in cycle i.

To elucidate the of role spatiotemporal factors in addition of those of classical discrete decision-making, we analyze in more detail the factors that determine the joint payoff of a dyad, using as a basis the observed trajectories of the dyads (Fig. 6). The joint payoff R equals the total number of target collections N times the payoff per target $r = 7$ cent

$$R = rN. \tag{25}$$

The total number of target collections N can be approximated by dividing the duration of each block $T = 20$ minutes by the mean collection cycle duration. The latter is the sum of the mean acquisition period duration $\langle t^{\text{acq}} \rangle$ and the constant collection period duration $t^{\text{col}} = 1$ second:

$$R = rN = r\frac{T}{\langle t^{\text{acq}} \rangle + t^{\text{col}}} + e_1. \tag{26}$$

The approximation error is denoted as e_1 and negligible ($r = 0.99, p < 10^{-6}$). The duration of an acquisition period t_i^{acq} equals the length of the so-called *limiting trajectory length* l_i divided by the corresponding speed on this trajectory s_i:

$$t_i^{\text{acq}} = \frac{l_i}{s_i}. \tag{27}$$

The length of the limiting trajectory l_i is either equal to the trajectory length of agent A or agent B. In case of a single target collection, it is that of the collecting agent, and in case of a joint target collection, it is that of the agent that entered last:

$$l_i = \begin{cases} l_i^A & \text{if } \vec{s}_i \text{ collected by agent } A, \\ l_i^B & \text{if } \vec{s}_i \text{ collected by agent } B, \\ l_i^A & \text{if } \vec{j}_i^X \text{ collected and agent } A \text{ entered last}, \\ l_i^B & \text{if } \vec{j}_i^X \text{ collected and agent } B \text{ entered last}. \end{cases} \tag{28}$$

By approximating the mean acquisition period duration $\langle t^{\text{acq}} \rangle$, we show that the joint payoff R is approximately proportional to the mean speed on and mean length of the limiting trajectory:

$$R = rN = r\frac{T}{\langle t^{\text{acq}} \rangle + t^{\text{col}}} + e_1 = r\frac{T}{\frac{\langle l \rangle}{\langle s \rangle} + t^{\text{col}}} + e_2. \tag{29}$$

The increased error, quantified by $e_2 > e_1$, originates from the inequality of the mean of a ratio to the ratio of means $\langle x/y \rangle \neq \langle x \rangle / \langle y \rangle$. Despite the increase, the error in predicting the joint payoff R remains negligible ($r = 0.99, p < 10^{-6}$). To decompose the joint payoff R further, we denote that the length of the limiting trajectory l is the sum of the excess length due to curvature l_i^C and the so-called *limiting distance* d_i.

$$l_i = l_i^C + d_i \tag{30}$$

The limiting distance d_i is the straight-line distance from the initial position of the limiting trajectory to the collected target. The limiting distance d_i results out of the position of the previously collected target and the amount of advantageous agent placement. Therefore, the limiting distance d_i is equal to the distance from the position of the previously collected target to the position of the subsequently collected target d_i^T minus the distance reduction due to advantageous placement d_i^R (see section 1.1)

$$l_i = l_i^C + d_i = l_i^C + d_i^T - d_i^R. \tag{31}$$

Substituting this into our joint payoff estimate, we observe its dependence on four variables:

$$R = rN = r\frac{T}{\langle t^{\text{acq}} \rangle + t^{\text{col}}} + e_1 = r\frac{T}{\frac{\langle l \rangle}{\langle s \rangle} + t^{\text{col}}} + e_2 = r\frac{T}{\frac{\langle l^C \rangle + \langle d^T \rangle - \langle d^R \rangle}{\langle s \rangle} + t^{\text{col}}} + e_2. \tag{32}$$

Here the error e_2 remains unchanged as the means of equal sample sizes are added. Two of the four variables (d^T and d^R) could in principle result out of classical discrete decision-making. The other two (l^C and s), however, result out of continuous, spatiotemporal interactions. Altogether, they shape the joint payoff in the cooperation–competition foraging game.

1.5 Individual payoffs

After analysing the joint payoff's dependencies, we proceed to decompose the individual payoff of a generic agent X and its counterpart, agent Y. We begin with two key relationships. We

Table S4 | Variables used in section 1.5, and their descriptions.

Variable	Description
X, Y	Generic agent names, could be either the blue or the orange target. If a variable is defined for agent X, the definition for agent Y is analogous.
R^X	Individual payoff of agent X.
R^X_Δ	Payoff difference of agent X to the other agent Y. Note that $R^X_\Delta = -R^Y_\Delta$.
n^X_s	Number of single target collections by agent X.
n^X_j	Number of joint target collections with a higher payoff share for agent X.
r_s	Payoff for a single target collection (7 cents).
$\hat{r}_j$	Higher payoff share of a joint target collection (5 cents).
$\check{r}_j$	Lower payoff share of a joint target collection (2 cents).
e	Error of predicting the payoff difference using only the single target difference. It is equal to the payoff difference due to differences in joint target type collections.

utilize (1) that the half of the joint payoff $R/2$ is exactly the within-dyad mean agent's individual payoffs (R^X and R^Y)

$$\frac{R^X + R^Y}{2} = \frac{R}{2} \tag{33}$$

and (2) that the difference from this intermediate payoff $R/2$ to the individual payoff R^X of an agent X equals the half of the inter-agent payoff difference $R^X_\Delta/2$ (defined later)

$$R^X = \frac{R}{2} + \frac{R^X_\Delta}{2}. \tag{34}$$

We observe that the individual payoff R^X depends on the payoff difference R^X_Δ and on the joint payoff R. From the previous section, we know on what variables the joint payoff R depends. Subsequently, we here analyze which variable is responsible for the inter-agent payoff difference

$$R^X_\Delta = R^X - R^Y. \tag{35}$$

The individual payoffs can be decomposed by the type of target:

$$R^X = n^X_s r_s + n^X_j \hat{r}_j + n^Y_j \check{r}_j \tag{36}$$

and

$$R^Y = n^Y_s r_s + n^Y_j \hat{r}_j + n^X_j \check{r}_j, \tag{37}$$

where n^X_s is the number of single target collections of agent X and $r_s = 7$ cent the payoff for a single target collection. n^X_j is the number of collected joint targets with a higher payoff share for agent X. This higher payoff share is $\hat{r}_j = 5$ cent. The lower joint target payoff share $\check{r}_j$ is 2 cents. The variables for agent Y are defined analogously. By substituting these calculations into our payoff difference formula we obtain:

$$R^X_\Delta = R^X - R^Y = (n^X_s - n^Y_s)r_s + (n^X_j - n^Y_j)(\hat{r}_j - \check{r}_j). \tag{38}$$

The right hand side of this equation has two components. The first, $(n^X_s - n^Y_s)r_s$, accounts for the payoff difference due to differences in single target collections. The second component, $(n^X_j - n^Y_j)(\hat{r}_j - \check{r}_j)$, accounts for the payoff difference due to difference in joint target collections. By leaving out the latter and introducing an error term e, we approximate the payoff difference

$$R^X_\Delta = R^X - R^Y = (n^X_s - n^Y_s)r_s + e \tag{39}$$

only with the difference in single targets. By demonstrating that the error e is negligible, we confirm that the inter-agent payoff difference is shaped predominantly by the difference in single targets (as visualized in Fig. 7b, $r = 0.99, p < 10^{-6}$). Thus, the individual payoff

R^X depends on the four variables known from previous section and on a fifth variable, the difference in single target collections between the agents.

1.6 Estimating the optimal strategy and cost of cooperation

Table S5 | Variables used in section 1.6, and their descriptions.

Variable	Description
Φ	Fraction of single targets (FST).
$\tilde{S}^X_\Delta$	Sensorimotor skill difference. It is the normalized single target difference S^X_Δ, considering only single target collections that are not due to defections like the "Different targets" and "Failed invitation" trajectory classes.
S^X_Δ	Normalized single target difference. It represents the difference in single target collections between agent X and agent Y, divided by the total number of single target collections. Note that $S^X_\Delta = -S^Y_\Delta$.
$\hat{R}^X(\Phi, \tilde{S}^X_\Delta)$	Estimated individual payoff of agent X given Φ and $\tilde{S}^X_\Delta$.
$\hat{R}(\Phi)$	Estimated joint payoff given Φ.
$\hat{R}^X_\Delta(\Phi, \tilde{S}^X_\Delta)$	Estimated inter-agent payoff difference for agent X given Φ and $\tilde{S}^X_\Delta$. Note that $\hat{R}^X_\Delta(\Phi, \tilde{S}^X_\Delta) = -\hat{R}^Y_\Delta(\Phi, \tilde{S}^Y_\Delta)$.
$\hat{\Phi}^X(\tilde{S}^X_\Delta)$	Estimated fraction of single targets of the optimal strategy of agent X given their skill difference $\tilde{S}^X_\Delta$.
$C(\Phi, \tilde{S}^X_\Delta)$	Estimated cost of cooperation for the higher-skilled agent X. It is called the cost of cooperation since the optimal strategy for a sufficiently higher-skilled agent is pure competition ($\Phi = 1$).

We show that participants are more cooperative than optimal and pay an associated cost of cooperation — the loss of income because of the choice of a non-optimal, overly cooperative, strategy. To estimate this cost of cooperation we estimate for each individual agent X the optimal strategy taking into account their estimated relative skill level. This optimal strategy is the strategy that maximizes the agent X's expected individual payoff. To predict this expected individual payoff, we model the individual payoff R^X of agent X as a function of FST Φ and skill difference $\tilde{S}^X_\Delta$ (defined later) analogous to the formula in section 1.5:

$$\hat{R}^X(\Phi, \tilde{S}^X_\Delta) = \frac{\hat{R}(\Phi)}{2} + \frac{\hat{R}^X_\Delta(\Phi, \tilde{S}^X_\Delta)}{2}, \tag{40}$$

where we use (1) an estimate of the inter-agent payoff difference $\hat{R}^X_\Delta(\Phi, \tilde{S}^X_\Delta)$ and (2) an estimate of the joint payoff $\hat{R}(\Phi)$. The latter follows out of an estimate of the number of target collections $\hat{N}(\Phi)$ which is analogous to that in section 1.4

$$\hat{R}(\Phi) = r\hat{N}(\Phi) = r\frac{T}{\frac{\hat{l}^C(\Phi)+\hat{d}^T(\Phi)-\hat{d}^R(\Phi)}{\hat{s}(\Phi)} + t^{\mathrm{col}}}. \tag{41}$$

The four dependencies, $\hat{l}^C(\Phi), \hat{d}^T(\Phi), \hat{d}^R(\Phi)$ and $\hat{s}(\Phi)$, of the joint payoff R are here estimated by fitting low-degree polynomials. This is a modeling choice to obtain later on the expected cost of cooperation for the average dyad. Alternatively, one could use here the values and assumptions from section 1.1 to obtain the cost of not pursuing the optimal dyad strategy. Having now defined the estimate for the joint payoff $\hat{R}(\Phi)$ we now continue with the estimate of the inter-agent payoff difference $\hat{R}^X_\Delta(\Phi, S^X_\Delta)$. Therefore, reusing the estimate from section 1.5:

$$R^X_\Delta = r_s(n^X_s - n^Y_s) + e \tag{42}$$

by multiplying $1 = \frac{N\Phi}{n_s^X + n_s^Y}$ we obtain

$$R_\Delta^X = r_s N \Phi \frac{n_s^X - n_s^Y}{n_s^X + n_s^Y} + e, \tag{43}$$

which motivates the definition of the normalized single target difference

$$S_\Delta^X = \frac{n_s^X - n_s^Y}{n_s^X + n_s^Y}. \tag{44}$$

This measure quantifies the efficiency of agent X in contrast to that of agent Y to collect single targets independently of FST. We only include single target collections when both agents moved straight to the single target ("concurrent" or "one ahead" to the same target), excluding defections ("Different targets" and "Failed invitation" trajectory classes from section 1.3) to obtain the competitive sensorimotor *skill difference* $\tilde{S}_\Delta^X$. This also makes this measure generalizable to different FST values. Now we define our new estimate of the inter-agent payoff difference as

$$\hat{R}_\Delta^X(\Phi, \tilde{S}_\Delta^X) = r\hat{N}(\Phi)\Phi\tilde{S}_\Delta^X, \tag{45}$$

where we utilize again our estimate of the number of target collections $\hat{N}(\Phi)$. Combining our estimate of the inter-agent payoff difference $\hat{R}_\Delta^X(\Phi, \tilde{S}_\Delta^X)$ with that of the joint payoff $\hat{R}(\Phi)$ we estimate agent X's individual payoff $\hat{R}^X(\Phi, \tilde{S}_\Delta^X)$ with high accuracy ($r = 0.88, p < 10^{-6}$) given only the FST Φ and the skill difference $\tilde{S}_\Delta^X$ (see Fig. 7c). Now we can estimate the FST value of agent X's optimal strategy

$$\hat{\Phi}^X(\tilde{S}_\Delta^X) = \arg\max_\Phi \hat{R}^X(\Phi, \tilde{S}_\Delta^X) \tag{46}$$

as well as the cost of cooperation of the higher-skilled agent X

$$C(\Phi, \tilde{S}_\Delta^X) = \max_\Phi(\hat{R}^X(\Phi, \tilde{S}_\Delta^X)) - \hat{R}^X(\Phi, \tilde{S}_\Delta^X) \tag{47}$$

for a given skill difference $\tilde{S}_\Delta^X$ (Fig. 7d).

Supplementary Figures

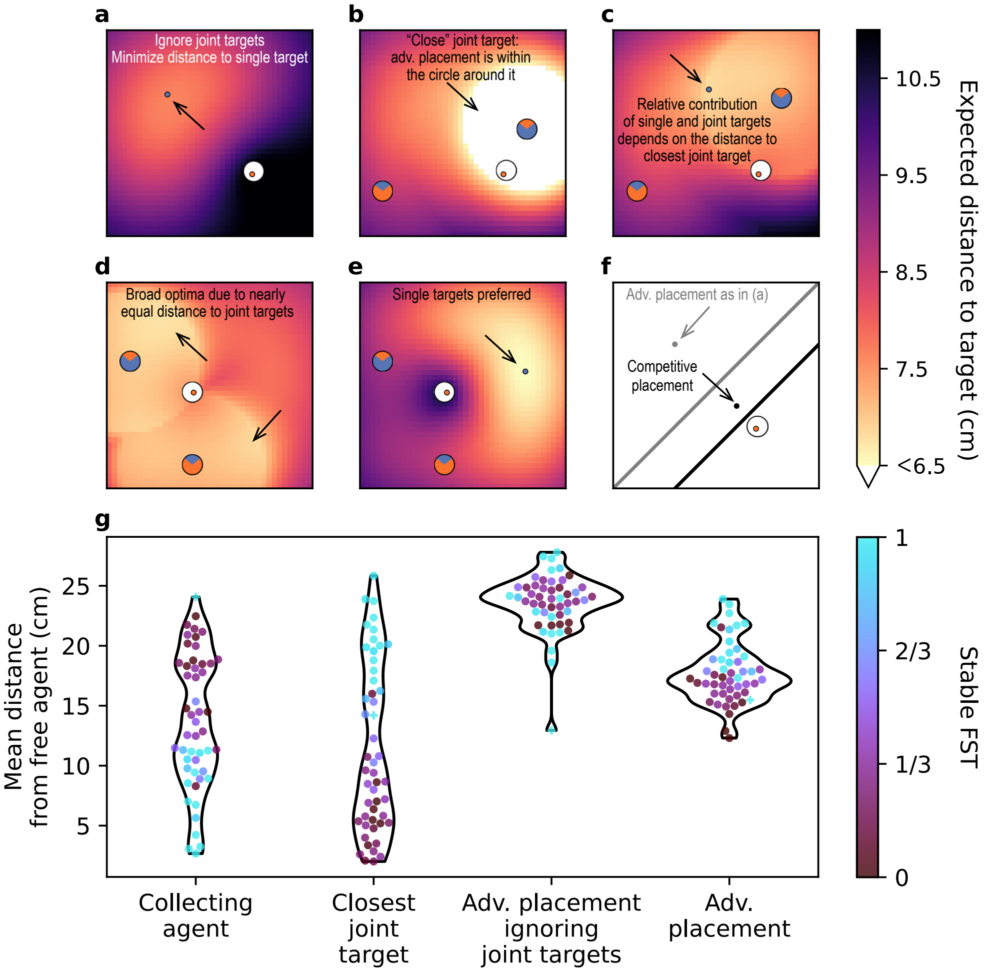

Fig. S1 | Advantageous and competitive placement, and actual placement during single target collections in each dyad. (**a-e**) Examples illustrating optimal (advantageous) placement of the non-collecting agent (blue dot, arrows) to minimize the expected distance to the next collected target (colormap). (**a**) Assuming no joint targets, the advantageous placement is on the other half of the game field, splitting the game field as much as possible between the two agents. Note that in (b-d), an equal weighting of target types is assumed (w=0.5). (**b**) When considering joint targets, the advantageous placement is within a circle centered on the closest joint target and extending to the currently collecting agent. (**c**) When the closest joint target is further away, the contribution of the single target becomes more apparent: the circle around the joint target is non-uniform and includes the optimum location splitting the game field between the two agents, similarly to (a). (**d**) In some spatial configurations, large parts of the game field are near optimal. (**e**) For a FST=1 dyad (w=0.99), the optimal placement ignores the joint targets (as in (a)). (**f**) Competitive placement example. The black dot represents a competitive placement, where the free, non-collecting agent optimizes the partitioning of the game field such that their probability of getting the next single target is maximized. For comparison, the gray dot represents the non-competitive advantageous placement as in (a). The gray line indicates the corresponding split of the game field into two parts, and the black line — the competitive split. (**g**) Actual mean distance from the free, non-collecting agent to four locations: (i) that of the collecting agent, (ii) that of the closest joint target, (iii) to the advantageous placement when ignoring the joint targets (w=0.99), and (iv) the advantageous placement (w=0.5). Instead of performing advantageous placement, agents in FST ≥ 0.9 dyads mainly perform competitive placement by placing themselves close to the collecting agent (as illustrated in (f)), and agents in $0.1 <$ FST < 0.9 dyads mostly place themselves next to the closest joint target. Note that FST ≤ 0.1 dyads are excluded from this analysis due to absent or very low number of single target collections.

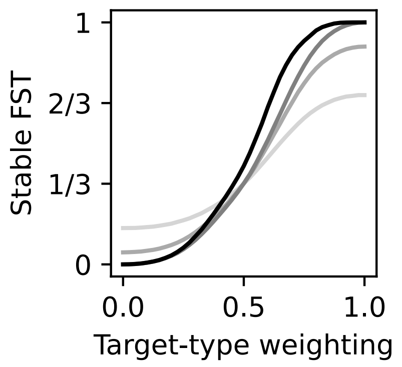

Fig. S2 | Cooperation–competition weighting of distances. To achieve different FSTs (fraction of single targets), the simulation requires different weightings of distances w reflecting specific dyads' preferences for selecting single targets vs. joint targets (eq. (3)). The black curve assumes an optimal "advantageous" placement of the non-collecting agent when the partner collects the single target, the dark gray curve assumes that the agents always move together and always select nearest (weighted) target, and the lighter gray lines assume non-optimal agents who move together but select a target randomly in 10% or 30% of trials (corresponding to the light gray lines in Fig. 3d).

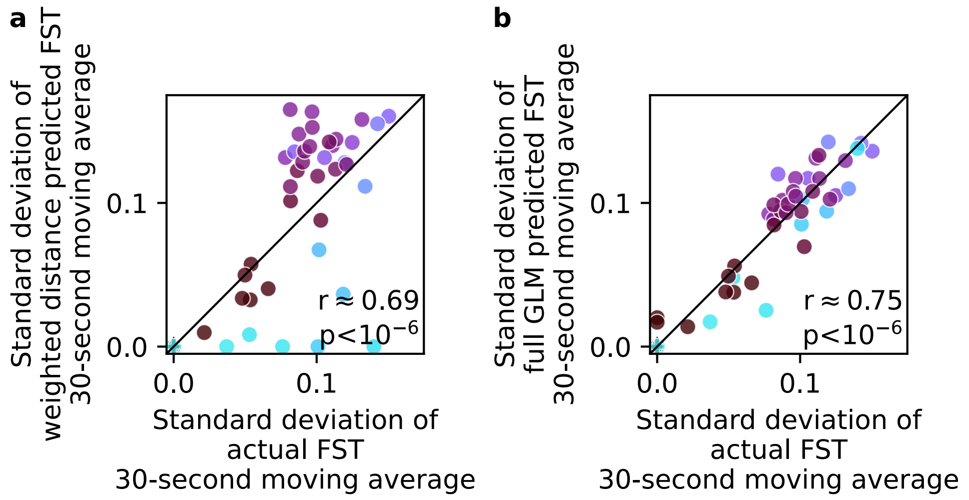

Fig. S3 | Variance of model predictions. (**a**) For high FST (light blue dots), the variance of predictions of the weighted distance model is too low whereas it is too high for intermediate FST (purple dots). (**b**) The full GLM is not only more accurate in its predictions (Fig. 4a), but also the variance of the predicted target type timecourse is better correlated with actual variance (Wilcoxon signed-rank test comparing the differences of standard deviations between 30-second moving average FST of the actual and each of the two model's predictions for the 40/58 dyads that exhibit FST fluctuations, $W = 258, p < 0.05, n = 40$, $\text{Mdn}_1 = -0.01\ [-0.03, 0.02]$, $\text{Mdn}_2 = 0.0008\ [-0.009, 0.02], r_{rb} = 0.32, CI = [0.04, 0.62]$).

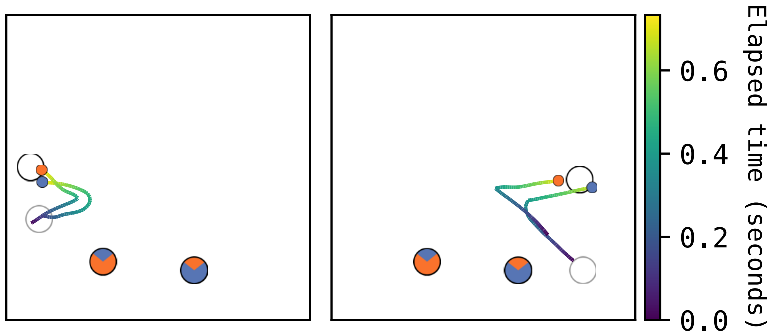

Fig. S4 | "Go-before-you-know" effect. Two example trajectories of a FST=1 dyad. Despite the certainty of target choice, the trajectories are strongly curved. Immediately after previous target collection, the agents move in the direction that minimizes the expected distance to the next target (the center of the game field), reflecting a preemptive strategy. Subsequently, once the newly appeared target is perceived, the trajectory is adjusted.

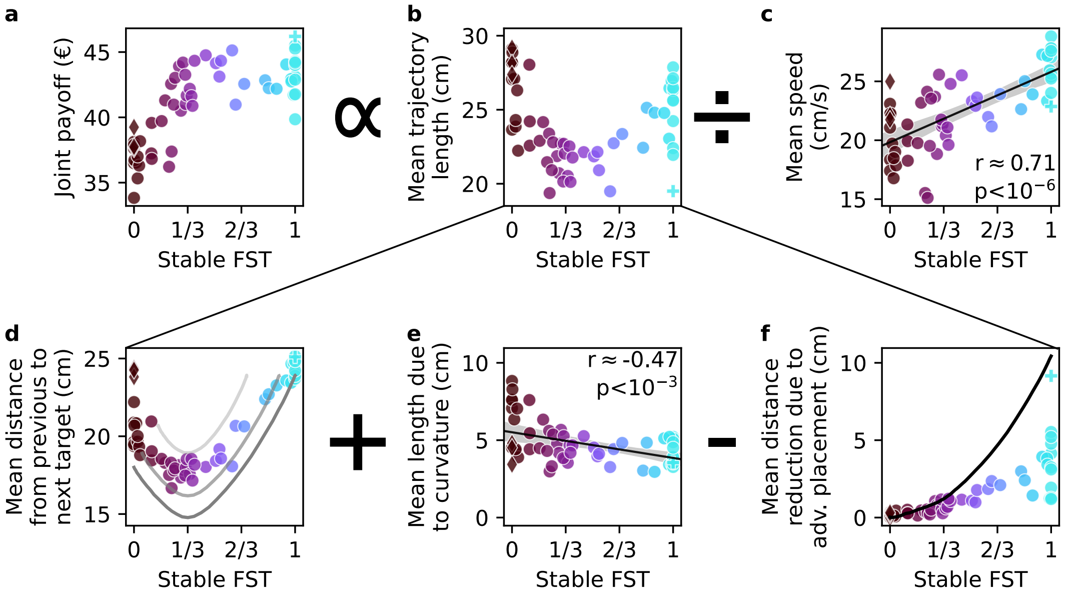

Fig. S5 | Spatiotemporal factors shape the payoff in a continuous action space. Note: panels (a), (b) and (c) are the same as in Fig. 6. (**a**) The joint payoff across both participants in a dyad is proportional to the mean acquisition duration. The mean acquisition duration is well-approximated by dividing the mean trajectory length (**b**) by the mean movement speed (**c**) on these trajectories. Note the increase in speed with higher fractions of single targets (FST). (**d-f**) We subdivide the mean trajectory length in (b) into three components. (**d**) Without advantageous placement, the trajectory length is at least the mean distance from the previous to the next target. The dark gray curve indicates the minimal mean distance attainable for each FST value, the lighter curves indicate the same path-minimizing strategy applied only in 90% and 70% of collection cycles. The highest distances are those of the three turn-taking dyads (diamond markers) who alternated between the two joint targets regardless of the distance. (**e**) Curved trajectories increase the mean trajectory length, especially at low FST because of frequent initial miscoordination between participants ($r(56) = -0.47, p < 10^{-3}, CI = [-0.65, -0.24]$. The exception to this pattern were the three turn-taking dyads, who eliminated miscoordination through their consistent strategy. (**f**) Advantageous placement reduces the trajectory length. To achieve this, the free agent must place itself strategically during a single target collection by the other. One dyad (plus marker) nearly reached the maximal attainable distance reduction (black curve, *cf.* Fig. 3d), using a *cooperative*, or at least a conflict-avoiding strategy, dividing the game field into a lower and upper half where each agent respectively collected the single targets.

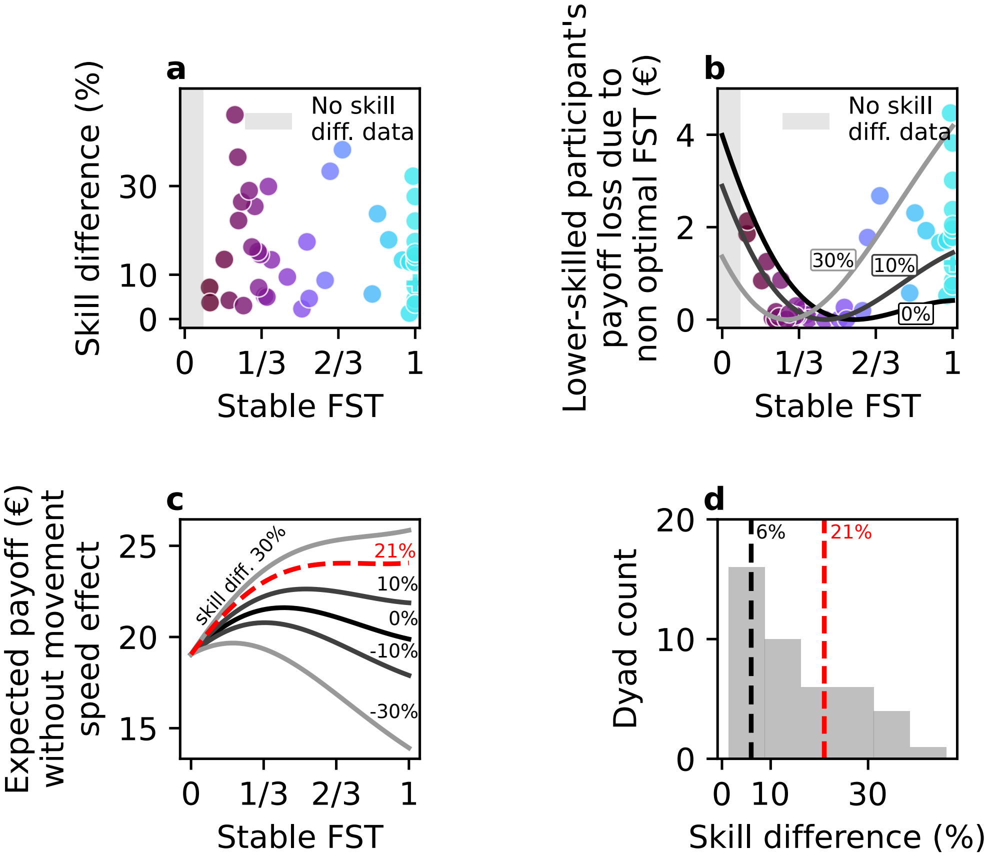

Fig. S6 | Skill differences and movement speed effect. (**a**) There is no observed relationship between skill difference in the dyad and the strategy they converged to. (**b**) Often the chosen strategy is also not optimal for the lower-skilled participant (*cf.* Fig. 7d). Note that each participant can increase FST on its own but decrease it only with the compliance of the other. (**c**) Expected payoff without the movement speed effect visualized as in Fig. 7c. The critical value of skill difference at which competition is optimal for the higher-skilled participant without taking into account the movement speed effects is at 21% (red dashed line). (**d**) Comparing the critical skill difference values expected with the movement speed effect (black dashed line) and without the speed effect (red dashed line). Due to the movement speed effect, competition is the best strategy for the majority of higher skilled participants.

Supplementary Movies

[YouTube playlist] | [Open Science Framework movies]

1. **Supplementary Movie S1:** YouTube or OSF
   Setup and game demonstration. Human Dyadic Interaction Platform setup and the game demonstration (60 s), followed by the replay of an example intermediate dyad.

2. **Supplementary Movie S2:** YouTube or OSF
   Cooperative example. Replay of a representative dyad from the cooperative group.

3. **Supplementary Movie S3:** YouTube or OSF
   Intermediate strategy example. Replay of a representative dyad from the intermediate group.

4. **Supplementary Movie S4:** YouTube or OSF
   Competition example. Replay of a representative dyad from the competitive group.

5. **Supplementary Movie S5:** YouTube or OSF
   Invitations examples. Collection cycles where one agent invites the other to a joint target.

6. **Supplementary Movie S6:** YouTube or OSF
   Cooperative turn-taking. Replay of one of three dyads who alternated between the two joint targets.

7. **Supplementary Movie S7:** YouTube or OSF
   Strongly curved trajectories. Examples of collection cycles featuring strongly curved trajectories, reflecting initial miscoordination and changes of mind.

8. **Supplementary Movie S8:** YouTube or OSF
   Competitive placement example. Replay of a dyad performing competitive advantageous placement.

9. **Supplementary Movie S9:** YouTube or OSF
   Cooperative placement for single targets. A special dyad that achieved nearly optimal advantageous placement by splitting the game field.

Instructions for participants

Welcome, and thank you for agreeing to participate in our experiment!

In this experiment you will play a game with another person through a transparent display.

Explanation of the game
In this game you need to collect targets to earn money. At the beginning the experimenter will assign a color to each player (blue and orange). On the screen you will see your own and other player's cursor (small circle colored respectively); you will be able to control your cursor with a computer mouse. The speed of the cursor is limited and by moving the mouse too fast you will have less control over it. To collect a target you need to place the cursor over the target and wait till the target completely disappears. At each time point of the game there will be 3 different targets available on the screen:

1. One white
2. One blue with a share of orange
3. One orange with a share of blue

You can collect white targets on your own — if you are first to select such a target, it becomes unavailable for the other player. To collect the colorful targets you need the other player to select the same target. Each collected target gives you a specific amount of money:

- White target gives 7 cents to the player who got it first
- Blue-orange target gives 5 cents to the blue player and 2 cents to the orange player
- Orange-blue target gives 5 cents to the orange player and 2 cents to the blue player

The amount of money you have collected will be displayed on the right side of the game field.

Structure of the experiment
The experiment will take ca. 2.5 hours in total. First, you will have a short game-tutorial to try out cursor control and target collection. Then, you will play 2 blocks, each 20 minutes, and between the blocks you will have an opportunity to take a break. From the start till the end of the experiment (including the breaks) we ask you to not communicate with the other player. In the end we will ask you to fill out a questionnaire.

Payment
You will receive the money you have collected in one of the blocks. In the end of the experiment, you will roll a dice to randomly select a block accordingly to which you will be paid:

- 1, 2, 3 - first block
- 3, 4, 5 - second block
