# Supplementary material for: Continuous dynamics of cooperation and competition in social decision-making
Source: Commun Psychol. 2025 Nov 24;3:170. doi: 10.1038/s44271-025-00348-w (PMC12644803; doi:10.1038/s44271-025-00348-w)
Supplement: Supplementary file 3 — Description of Additional Supplementary Files [file 44271_2025_348_MOESM3_ESM.pdf]

Description of Additional Supplementary Files

File name- Supplementary Movie S1

File description – Setup and game demonstration. Human Dyadic Interaction Platform setup and the game demonstration (60 s), followed by the replay of an example intermediate dyad.

File name- Supplementary Movie S2

File description – Cooperative example. Replay of a representative dyad from the cooperative group.

File name- Supplementary Movie S3

File description – Intermediate strategy example. Replay of a representative dyad from the intermediate group.

File name- Supplementary Movie S4
File description – Competition example. Replay of a representative dyad from the competitive group.

File name- Supplementary Movie S5
File description – Invitations examples. Collection cycles where one agent invites the other to a joint target.

File name- Supplementary Movie S6
File description – Cooperative turn-taking. Replay of one of three dyads who alternated between the two joint targets.

File name- Supplementary Movie S7

File description – Strongly curved trajectories. Examples of collection cycles featuring strongly curved trajectories, reflecting initial miscoordination and changes of mind.

File name- Supplementary Movie S8

File description – Competitive placement example. Replay of a dyad performing competitive advantageous placement

File name- Supplementary Movie S9
File description – Cooperative placement for single targets. A special dyad that achieved nearly optimal advantageous placement by splitting the game field.
