# Supplementary material for: Continuous dynamics of cooperation and competition in social decision-making
Source: Commun Psychol. 2025 Nov 24;3:170. doi: 10.1038/s44271-025-00348-w (PMC12644803; doi:10.1038/s44271-025-00348-w)
Supplement: Supplementary file 13 — Reporting Summary [file 44271_2025_348_MOESM13_ESM.pdf]

nature portfolio

Corresponding author(s): Igor Kagan

Last updated by author(s): YYYY-MM-DD

Reporting Summary

Nature Portfolio wishes to improve the reproducibility of the work that we publish. This form provides structure for consistency and transparency in reporting. For further information on Nature Portfolio policies, see our Editorial Policies and the Editorial Policy Checklist.

Statistics

For all statistical analyses, confirm that the following items are present in the figure legend, table legend, main text, or Methods section.

n/a	Confirmed	
☐	☒	The exact sample size (*n*) for each experimental group/condition, given as a discrete number and unit of measurement
☐	☒	A statement on whether measurements were taken from distinct samples or whether the same sample was measured repeatedly
☐	☒	The statistical test(s) used AND whether they are one- or two-sided<br>*Only common tests should be described solely by name; describe more complex techniques in the Methods section.*
☒	☐	A description of all covariates tested
☐	☒	A description of any assumptions or corrections, such as tests of normality and adjustment for multiple comparisons
☐	☒	A full description of the statistical parameters including central tendency (e.g. means) or other basic estimates (e.g. regression coefficient) AND variation (e.g. standard deviation) or associated estimates of uncertainty (e.g. confidence intervals)
☐	☒	For null hypothesis testing, the test statistic (e.g. *F*, *t*, *r*) with confidence intervals, effect sizes, degrees of freedom and *P* value noted<br>*Give P values as exact values whenever suitable.*
☒	☐	For Bayesian analysis, information on the choice of priors and Markov chain Monte Carlo settings
☒	☐	For hierarchical and complex designs, identification of the appropriate level for tests and full reporting of outcomes
☐	☒	Estimates of effect sizes (e.g. Cohen's *d*, Pearson's *r*), indicating how they were calculated

Our web collection on statistics for biologists contains articles on many of the points above.

Software and code

Policy information about availability of computer code

Data collection	Data collection was conducted using a custom Python 3.10 code, available at https://gitlab.gwdg.de/darius.lewen/lewen_et_al, and linked to at https://osf.io/56hw7/
Data analysis	Data analysis was conducted using a custom Python 3.10 code and R version 4.4.2, available at https://gitlab.gwdg.de/darius.lewen/lewen_et_al, and linked to at https://osf.io/56hw7/

For manuscripts utilizing custom algorithms or software that are central to the research but not yet described in published literature, software must be made available to editors and reviewers. We strongly encourage code deposition in a community repository (e.g. GitHub). See the Nature Portfolio guidelines for submitting code & software for further information.

Data

Policy information about availability of data

All manuscripts must include a data availability statement. This statement should provide the following information, where applicable:

- Accession codes, unique identifiers, or web links for publicly available datasets
- A description of any restrictions on data availability
- For clinical datasets or third party data, please ensure that the statement adheres to our policy

All data collected in the experiment and used for the analyses is available at https://osf.io/56hw7/ and https://gitlab.gwdg.de/darius.lewen/lewen_et_al

Research involving human participants, their data, or biological material

Policy information about studies with human participants or human data. See also policy information about sex, gender (identity/presentation), and sexual orientation and race, ethnicity and racism.

Reporting on sex and gender	The analyzed dataset included 116 participants, of whom 19 self-identified as female and the remainder as male. The predominance of male participants was due to recruitment overlap with a related ongoing study investigating hormonal effects in males. These participants responded affirmatively to the question “Bist du chromosomal geschlechtlich männlich?” (“Are you chromosomally male?”). No analyses based on sex or gender were conducted; therefore, the terms “sex” and “gender” are not used throughout the manuscript. Self-reported gender information is provided in the openly available dataset.
Reporting on race, ethnicity, or other socially relevant groupings	We did not collect or report data on participants’ race, ethnicity, or other socially defined groupings, as these variables were not pertinent to the research questions addressed in this study.
Population characteristics	Please see above and below.
Recruitment	Participants were recruited via student chat groups, ads, and flyers around the Goettingen campus, and were predominantly Bachelor, Master, or PhD German and international students. As such, the sample is biased toward individuals with a typical educational and socio-economic background for university students, likely characterized by higher education levels, middle to upper socio-economic status, and familiarity with academic settings. This may potentially limit the generalizability of the findings to broader or more diverse populations.
Ethics oversight	The experimental protocol was approved by the ethics committee of the Georg-Elias-Mueller-Institute for Psychology, University of Goettingen.

Note that full information on the approval of the study protocol must also be provided in the manuscript.

Field-specific reporting

Please select the one below that is the best fit for your research. If you are not sure, read the appropriate sections before making your selection.

☐ Life sciences ☒ Behavioural & social sciences ☐ Ecological, evolutionary & environmental sciences

For a reference copy of the document with all sections, see nature.com/documents/nr-reporting-summary-flat.pdf

Behavioural & social sciences study design

All studies must disclose on these points even when the disclosure is negative.

Study description	Quantitative experimental and computational modeling study, using continuous 2D cursor movement data from pairs of participants collecting cooperative and competitive targets in a foraging game in a real-time face-to-face environment.
Research sample	Predominantly Bachelor, Master or PhD German and international students, age range 18–36 years, 116 participants in 58 dyads (19 females, 97 males, mean ± SD age: 25 ± 4 years, range 18–36 years, 46 male dyads, 7 female dyads, 5 mixed female/male dyads). Most participants were male because they were recruited for a related ongoing study of male hormonal effects.
Sampling strategy	A convenience sampling strategy was employed to recruit approximately 60 dyads. As this was a novel experimental paradigm with no established effect sizes or outcome distributions, the target sample size was informed by previous research on social interaction and sensorimotor decision-making. The sample size was selected to balance practical feasibility with the goal of capturing a potentially broad range of emergent dyadic behaviors and interaction strategies.
Data collection	The experiments were conducted in the Dyadic Interaction Platform laboratory at the German Primate Center, as detailed in the Methods section. In brief, two participants sat face-to-face and played a visually guided Cooperation–Competition Foraging game. Each participant used a computer mouse to control movements of a cursor ("agent") within a 2D field on a large, bidirectional transparent OLED screen positioned between participants. An experimenter—either one of the study authors or a trained student assistant—initiated and supervised each session from a control console behind a partition, remaining out of direct view of the participants. The authors were fully informed about the study design and general goals and had real-time visual access to the interaction via a mirrored display. However, they had no means to influence participants’ behavior and were blind to the cumulative dynamics of dyadic strategies, which required offline analysis. The only real-time information available to them during the task was the ongoing accumulation of payoffs.
Timing	Data were collected between January 2022 and December 2022.
Data exclusions	As stated in the Methods, 4 out of 62 dyads were excluded from the current analysis because they exhibited large differences in behavior between blocks. The exclusion criterion was not pre-established but was developed during the initial analysis, with the goal to focus on stable behavioral patterns.

Non-participation	No participants dropped out.
Randomization	Participants were not allocated into experimental groups.

Reporting for specific materials, systems and methods

We require information from authors about some types of materials, experimental systems and methods used in many studies. Here, indicate whether each material, system or method listed is relevant to your study. If you are not sure if a list item applies to your research, read the appropriate section before selecting a response.

Materials & experimental systems

n/a	Involved in the study
☒	☐ Antibodies
☒	☐ Eukaryotic cell lines
☒	☐ Palaeontology and archaeology
☒	☐ Animals and other organisms
☒	☐ Clinical data
☒	☐ Dual use research of concern
☒	☐ Plants

Methods

n/a	Involved in the study
☒	☐ ChIP-seq
☒	☐ Flow cytometry
☒	☐ MRI-based neuroimaging

Plants

Seed stocks	N/A
Novel plant genotypes	N/A
Authentication	N/A
